# Impact of neoadjuvant immunotherapy combined with chemotherapy or chemoradiotherapy on postoperative safety in locally advanced esophageal squamous cell carcinoma: a propensity score-matched retrospective cohort study

**DOI:** 10.3389/fonc.2025.1573597

**Published:** 2025-05-21

**Authors:** Yixin Li, Gang Xiao, Bo Yang, Yidan Hong, Zeng Chen, Lingling Gu, Cheng Kong, Lijun Zhao, Zihao Zhu, Qicen Xu, Yu Chen, Ming Jiang, Xiangzhi Zhu, Ning Jiang

**Affiliations:** ^1^ Department of Radiation Oncology, The Affiliated Cancer Hospital of Nanjing Medical University, Jiangsu Cancer Hospital, Jiangsu Institute of Cancer Research, Nanjing, China; ^2^ Department of Anesthesiology, The Affiliated Cancer Hospital of Nanjing Medical University, Jiangsu Cancer Hospital, Jiangsu Institute of Cancer Research, Nanjing, China; ^3^ Department of Medical Imaging Center, The Affiliated Cancer Hospital of Nanjing Medical University, Jiangsu Cancer Hospital, Jiangsu Institute of Cancer Research, Nanjing, China; ^4^ Department of Information, The Affiliated Cancer Hospital of Nanjing Medical University, Jiangsu Cancer Hospital, Jiangsu Institute of Cancer Research, Nanjing, China; ^5^ Department of Thoracic Surgery, The Affiliated Cancer Hospital of Nanjing Medical University, Jiangsu Cancer Hospital, Jiangsu Institute of Cancer Research, Nanjing, China

**Keywords:** esophagectomy, esophageal squamous cell carcinoma (ESCC), neoadjuvant immunotherapy, perioperative complications, immune checkpoint inhibitors (ICIs)

## Abstract

**Introduction:**

The combination of immunotherapy with neoadjuvant chemotherapy (nICT) or chemoradiotherapy (nICRT) represents a novel treatment approach for patients with locally advanced esophageal squamous cell carcinoma (LA-ESCC). This study aimed to compare postoperative complications between patients who underwent esophagectomy directly and those who received surgery following neoadjuvant immunotherapy combining treatments (nIComT) including nICT or nICRT.

**Materials and methods:**

A retrospective analysis was conducted on patients with LA-ESCC at our center. A 1:1 propensity score matching (PSM) was used to eliminate baseline characteristics differences. The primary endpoint was postoperative complications, which were assessed based on the Esophageal Cancer Complications Consensus Group (ECCG) criteria, and the severity was evaluated according to the Clavien-Dindo classification.

**Results:**

After PSM, 116 matched patients were analyzed in both the surgery-alone and nIComT group. The overall complication rates between the two groups were similar (51.7% *vs* 56.0%, *P*=0.510). Incidence of cardiovascular complications, most of which were grade I and II, was increased in the nIComT group compared with the surgery-alone group(*P*=0.003). The higher rate of cardiovascular complications mainly due to hypotension (52.6% *vs* 42.2%, *P*=0.004) requiring intervention including the use of vasopressors, or transfusion. Additionally, more patients in the nIComT group received perioperative transfusion (34.5% *vs* 14.7%, *P*<0.001), as well as an extended operation duration (276 ± 66min *vs* 246 ± 63min, *P*<0.001), when compared to the surgery-alone group. The logistic regression analyses of potential risk factors for cardiovascular complications showed that receiving neoadjuvant treatment was independently associated with cardiovascular complications (OR=2.03, 95% CI=1.15-3.57, *P*=0.015).

**Conclusion:**

Our study highlights an increased risk of cardiovascular complications among patients received nIComT, underscoring the significance of postoperative circulatory interventions. Further prospective studies are needed for validation.

## Introduction

Esophageal cancer (EC) ranks as the eleventh most common malignancy and seventh in mortality worldwide (1). EC is particularly prevalent in Asia, where China accounting for over half of all cases (2). Esophageal squamous cell carcinoma (ESCC) represents the predominant subtype in China, comprising approximately 90% of EC cases (3). Neoadjuvant chemotherapy (nCT) or chemoradiotherapy (nCRT) combined with esophagectomy is the standard of care for locally advanced esophageal squamous cell carcinoma (LA-ESCC). In spite of this, the 5-year overall survival (OS) rate for LA-ESCC was 30.3% as of 2015 (4). Therefore, it is essential to explore new therapeutic strategies to enhance treatment efficacy and improve survival benefits for patients with LA-ESCC.

Immune checkpoint inhibitors (ICIs) have emerged as a promising therapeutic strategy for ESCC (5, 6). Building upon the established efficacy of ICIs as adjuvant therapy in LA-ESCC patients demonstrated in the CheckMate 577 trial (7), multiple prospective clinical trials have subsequently investigated the integration of ICIs with neoadjuvant chemotherapy (nICT) or chemoradiotherapy (nICRT) (8–11). Several studies have demonstrated pathological complete response (pCR) rates of 16.7%-39.2% in LA-ESCC patients who underwent nICT (9, 12, 13), while the NICE trial reported a 2-year OS rate of 78.1% (14). Notably, nICRT have shown a trend of enhanced efficacy compared to the CROSS and NEOCRTEC5010 trials (15, 16), with pCR rates ranging from 22.6% to 55.6% (8, 11, 17). In summary, nICT or nICRT represents a promising therapeutic for patients with LA-ESCC.

Although neoadjuvant immunotherapy has shown promise for LA-ESCC (8, 10, 11), the postoperative safety implications of combining ICIs with neoadjuvant therapy contentious. A meta-analysis revealed that, compared to nCT, nICT significantly increased surgery cancellation rates due to grade≥3 treatment-related adverse events (TRAEs) (18). Furthermore, neoadjuvant immunotherapy has been identified as an independent risk factor for increased surgical complexity (19). While limited evidence in LA-ESCC, the safety profile of combining immunotherapy with conventional neoadjuvant regimens requires urgent investigation.

This retrospective study compared postoperative safety outcomes between patients underwent esophagectomy directly and those received neoadjuvant immunotherapy combining treatments (nIComT), which include nICT or nICRT, aiming to provide clinical evidence for postoperative safety management in LA-ESCC.

## Materials and methods

### Study design

This is a retrospective clinical study screening consecutive patients with LA-ESCC, staged cT1-4aN+M0/cT3-4aN0M0 according to the AJCC 8^th^ edition (20), who underwent esophagectomy at our center between January 2020 to January 2022. The primary endpoint was postoperative safety. The secondary endpoint was perioperative outcomes.

This study was conducted according to the Declaration of Helsinki and received ethical approval from the Institutional Review Board and Ethics Committee of our center. The requirement for informed consent was waived due to the anonymity of the data.

### Patient selection

The inclusion criteria were as follows: (1) Eastern Cooperative Oncology Group (ECOG) performance status ≤1; (2) complete clinical data available; and (3) normal blood, liver and kidney functions. Patients who had undergone endoscopic submucosal dissection (ESD) for esophageal lesions, received other neoadjuvant therapy or relevant antitumor therapy for malignancies other than ESCC were excluded.

### Neoadjuvant treatment

By January 2022, all patients in the nIComT group had received 2–4 cycles of nICT prior to esophagectomy. The neoadjuvant immunotherapy regimen included five PD-1 inhibitors: sintilimab (200 mg, i.v, q3w), toripalimab (240 mg i.v, q3w), camrelizumab (200 mg i.v, q3w), pembrolizumab (200 mg i.v, q3w), and tislelizumab (200 mg i.v, q3w). The chemotherapy regimen consisted of paclitaxel (135–175 mg/m², i.v, q3w) plus carboplatin (area under the curve [AUC]=5, i.v, q3w) or cisplatin (75 mg/m², i.v, q3w) (TP regimen).

For patients received nICRT, the total radiation dose was 30 Gy, delivered as a short-course regimen in 12 fractions (2.5 Gy per fraction, 5 fractions weekly) under conventional fractionation. All cases were derived from a prospective clinical trial conducted at our center (SCALE-1 trial, ChiCTR2100045104) (17). The radiotherapy planning employed involved-field irradiation technique with gross tumor volume (GTV) encompassing primary esophageal lesions and metastatic lymph nodes (LNs) defined by radiographic criteria (≥10mm short-axis for non-special regions, ≥5mm for LNs in esophageal/tracheoesophageal groove areas, or presence of malignant features like central necrosis/ring enhancement/eccentric calcification). No clinical target volume (CTV) was defined. Planning target volume (PTV) margins were created by expanding GTV by 0.8cm for mid-upper thoracic lesions and 1.0cm for lower thoracic lesions, with additional 2.0cm longitudinal margin along esophageal axis. Lymph node PTVs were generated by 1.0cm isotropic expansion from GTV (17).

### Surgery

Surgery was performed within 4–8 weeks after the end of the last neoadjuvant treatment. All patients received the Ivor-Lewis operation (right transthoracic esophagectomy with reconstruction and laparoscopic dissection) or the McKeown operation (right thoracotomy, laparoscopy dissection, and left cervical esophagectomy with reconstruction) which are the usual procedures at our center and widely used in China. Esophagectomy and cervical or thoracic anastomosis were performed on all patients using gastric reconstruction of the esophagus. The right recurrent laryngeal nerve chain was fully dissected, but the left recurrent laryngeal nerve chain was only dissected in select patients with suspected metastatic lymph nodes.

### Outcomes measurement

Postoperative complications were evaluated using the Esophageal Cancer Complications Consensus Group (ECCG) criteria (21), including postoperative hypotension defined as mean arterial pressure (MAP) below 60 mmHg (22). Severity was assessed by the Clavien-Dindo classification, specifically addressing ≥grade III complications requiring surgical intervention, life-threatening conditions necessitating ICU readmission, and mortality (23). TRAEs during neoadjuvant therapy were graded using the National Cancer Institute Common Terminology Criteria for Adverse Events (version 5.0) (24), with immune-related adverse events (irAEs) defined as events like hepatitis, colitis, pneumonitis, and myocarditis connected to ICIs with potential immunologic causes (25, 26). Both TRAEs and irAEs were systematically evaluated in a double-blind manner by two senior radiation oncologists and two radiologists based on clinical and imaging data. Mortality was defined as postoperative death within 30 days.

### Statistical analysis

PSM was employed to eliminate baseline differences between the surgery-alone group and the nIComT group. Variables including age, body mass index (BMI), gender, ECOG performance status, comorbidities, smoking history, tumor location, cTNM stage, anastomotic site, surgical procedure, and extent of lymphadenectomy were incorporated into the PSM model construction using nearest matching with a caliper value of 0.02 and a 1:1 matching ratio (27, 28). Continuous variables were described as mean ± standard deviation (SD), with group comparisons made via Wilcoxon rank-sum test or *t-*test. Categorical variables were presented as frequencies or percentages and compared using Pearson chi-square test or Fisher’s exact test. Conditional logistic regression analysis was utilized to validate potential risk factors, where variables with *P*<0.20 in univariate analysis were included in multivariate analysis (29). All statistical tests were two-sided, with *P*<0.05 indicating significance. Data were analyzed using R version 4.3.1 and SPSS 27.0.

## Results

### Patient characteristics

**Figure 1:**
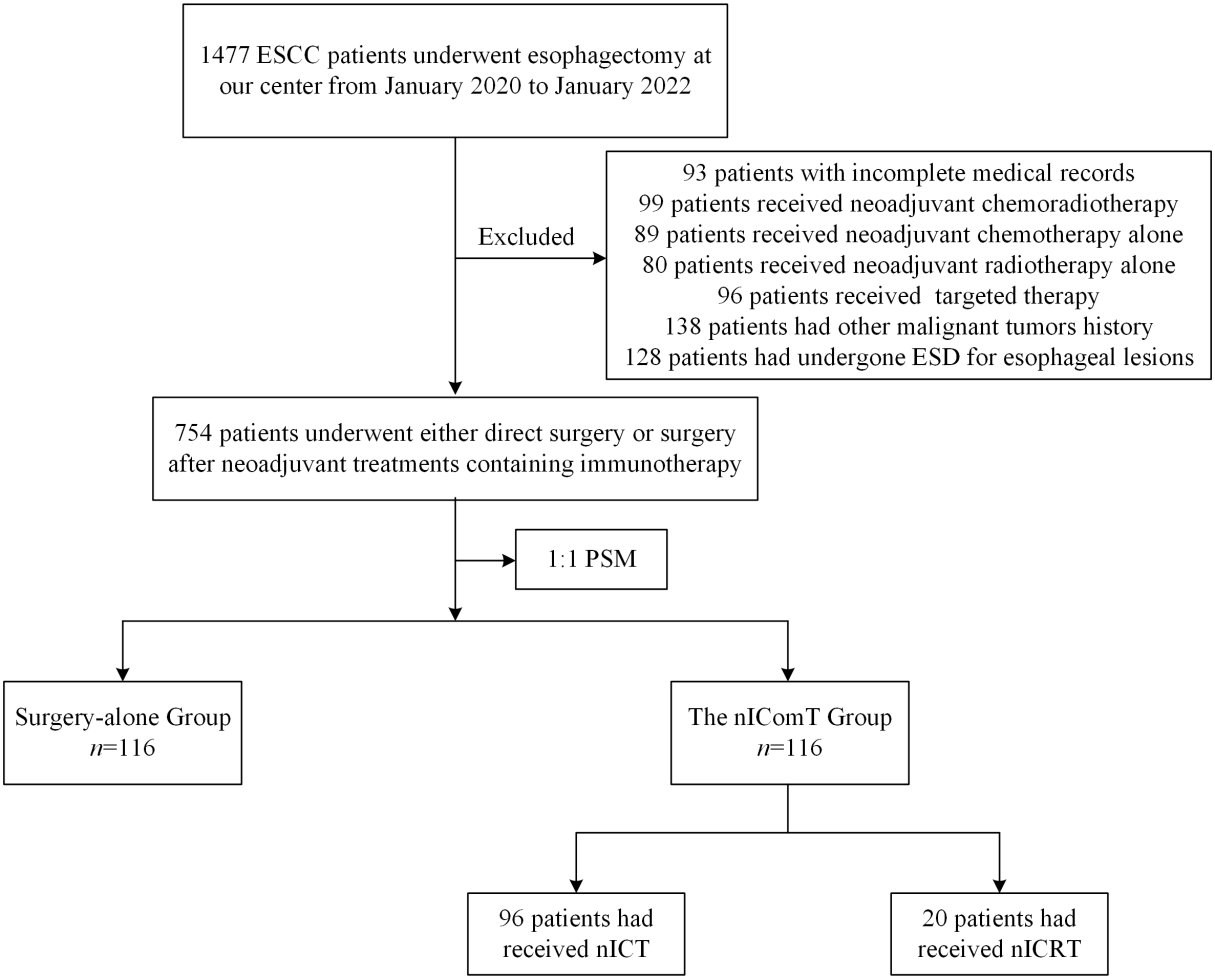
Patient selection flowchart. ESD, endoscopic submucosal dissection; PSM, propensity score matching; nICT, neoadjuvant immunotherapy combined with chemotherapy; nICRT, neoadjuvant immunotherapy combined with chemoradiotherapy.

**Figure 2:**
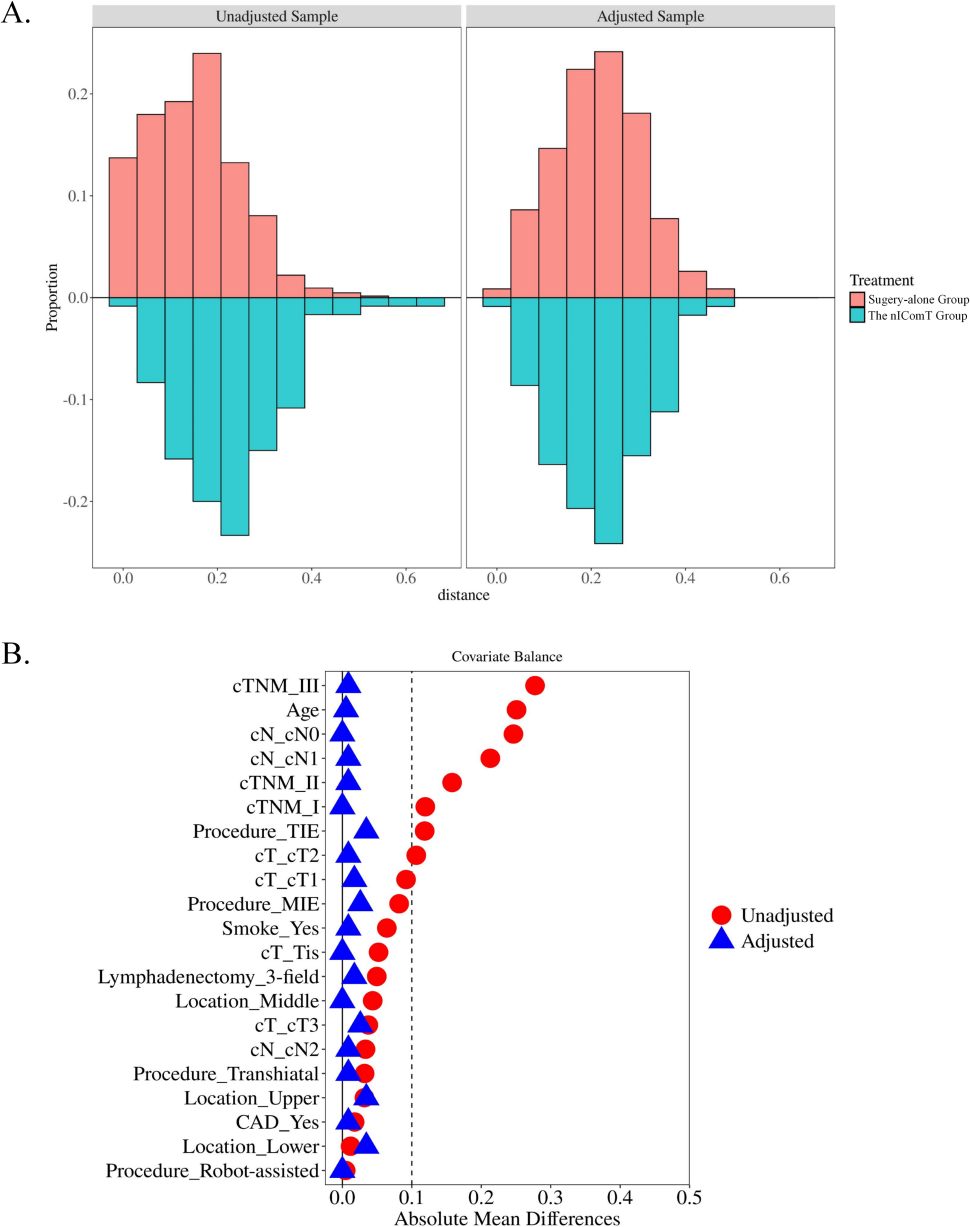
**(A)** Histogram showing the balance for the categorical variable; **(B)** Covariate balance measured by standardized mean difference. nIComT, neoadjuvant immunotherapy combination treatments; CAD, coronary artery disease; T, tumor; Tis, carcinoma *in situ*; N, node; TIE, transmediastinal esophagectomy; MIE, minimally invasive esophagectomy.

The patient screening process is shown in **Figure 1**. Between January 2020 and January 2022, a total of 1,477 patients underwent esophagectomy were screened at our center. After applying exclusion criteria, 723 patients were excluded, resulting in 754 eligible for study inclusion. Among eligible patients, 634 underwent esophagectomy directly, while 120 received nIComT prior to surgery. To minimize selection bias, a 1:1 PSM was performed between the surgery-alone and the nIComT groups. After PSM, each group comprised 116 patients. Within the nIComT group, 96 patients received nICT and 20 received nICRT. Baseline characteristics of patients before and after PSM are presented in **Table 1**, with features demonstrating balance between groups (*P*>0.05). Detailed comparisons of baseline characteristics before and after PSM adjustment are visualized in **Figure 2**.

**Table 1:** Baseline characteristics of patients before and after propensity score matching.

Patient characteristics	Before matching			SMD	After matching			SMD
	Surgery-alone group (*n*=634)	The nIComT group (*n*=120)	*P* value		Surgery-alone Group (*n*=116)	The nIComT group (*n*=116)	*P* value	
Age (mean ± SD)	65.70 ± 7.35	63.93 ± 6.80	.005	0.251	64.04 ± 7.60	64.08 ± 6.51	.970	0.005
BMI (mean ± SD)	23.27 ± 2.97	23.38 ± 2.96	.886	0.049	23.36 ± 3.05	23.36 ± 2.64	.566	0.082
Gender			.919	0.010			.749	0.041
Male	494 (77.9%)	93 (77.5%)			92 (79.3%)	90 (77.6%)		
Female	140 (22.1%)	27 (22.5%)			24 (20.7%)	26 (22.4%)		
ECOG			.467	0.074			.473	0.092
0	406 (64.0%)	81 (67.5%)			84 (72.4%)	79 (68.1%)		
1	228 (36.0%)	39 (32.5%)			32 (27.6%)	37 (31.9%)		
Comorbidity								
Hypertension	145 (22.9%)	28 (23.3%)	.912	0.011	17 (14.7%)	27 (23.3%)	.094	0.204
Diabetes	37 (5.8%)	9 (7.5%)	.485	0.063	3 (2.6%)	9 (7.8%)	.075	0.193
CAD	27 (4.3%)	3 (2.5%)	.516	0.113	2 (1.7%)	3 (2.6%)	1.000	0.054
Arrhythmia	33 (5.2%)	5 (4.2%)	.634	0.052	4 (3.5%)	5 (4.3%)	1.000	0.042
CI	18 (2.8%)	6 (5.0%)	.341	0.099	3 (2.6%)	6 (5.2%)	.497	0.117
Smoking history	104 (16.4%)	12 (10.0%)	.075	0.213	11 (9.5%)	12 (10.3%)	.826	0.028
Tumor location			.520	0.115			.544	0.075
Upper	73 (11.5%)	10 (8.3%)			6 (5.2%)	10 (8.6%)		
Middle	358 (56.5%)	73 (60.8%)			71 (61.2%)	71 (61.2%)		
Lower	203 (32.0%)	37 (30.8%)			39 (33.6%)	35 (30.2%)		
cT stage			<.001	0.587			.785	0.054
Tis	33 (5.2%)	0 (0.0%)			0 (0.0%)	0 (0.0%)		
cT1	74 (11.7%)	3 (2.5%)			5 (4.3%)	3 (2.6%)		
cT2	165 (26.0%)	44 (36.7%)			41 (35.3%)	40 (34.5%)		
cT3	362 (57.1%)	73 (60.8%)			70 (60.3%)	73 (62.9%)		
cN stage			<.001	0.616			.975	0.019
cN0	285 (45.0%)	24 (20.0%)			24 (20.7%)	24 (20.7%)		
cN1	312 (49.2%)	85 (70.8%)			80 (69.0%)	81 (70.0%)		
cN2	37 (5.8%)	11 (9.2%)			12 (10.3%)	11 (9.5%)		
cTNM Stage ^a^			<.001	1.314			1.000	0.021
I	81 (12.8%)	1 (0.8%)			0 (0.0%)	0 (0.0%)		
II	227 (35.8%)	24 (20.0%)			25 (21.6%)	24 (20.7%)		
III	326 (51.4%)	95 (79.2%)			93 (78.4%)	94 (79.3%)		
Anastomotic			.923	0.010			.128	0.195
Cervical	246 (38.8%)	46 (38.3%)			34 (29.3%)	45 (38.8%)		
Thoracic	388 (61.2%)	74 (61.7%)			82 (70.7%)	71 (61.2%)		
Procedure			.087	0.238			.855	0.052
TIE	263 (41.5%)	64 (53.3%)			64 (55.2%)	60 (51.7%)		
MIE	316 (49.8%)	50 (41.7%)			47 (40.5%)	50 (43.1%)		
Transhiatal	52 (8.2%)	6 (5.0%)			5 (4.3%)	6 (5.2%)		
Robot-assisted	3 (0.5%)	0 (0.0%)			0 (0.0%)	0 (0.0%)		
Lymphadenectomy			.034	0.165			.581	0.068
2-field	602 (95.0%)	108 (90.0%)			110 (94.8%)	108 (93.1%)		
3-field	32 (5.0%)	12 (10.0%)			6 (5.2%)	8 (6.9%)		

a The clinical stages were evaluated according to the criteria of the American Joint Committee on Cancer, eighth edition.

nIComT, neoadjuvant immunotherapy combination treatments; SMD, standardized mean difference; SD, standard deviation; BMI, body mass index; ECOG, Eastern Cooperative Oncology Group; CAD, coronary artery disease; CI, cerebral infarction; T, tumor; Tis, carcinoma *in situ*; N, node; TIE, transmediastinal esophagectomy; MIE, minimally invasive esophagectomy.

### Postoperative complications

**Table 2** presents the overall incidence of postoperative complications and rates of Clavien-Dindo grade ≥III complications in both groups. No significant difference was observed in total complication rates between the two groups (51.7% *vs* 56.0%, *P*=0.510). The incidence of complications in pulmonary, gastrointestinal, urologic, thromboembolic and neuropsychiatric systems, and infections was comparable between groups (*P*>0.05). However, the nIComT group showed a significantly higher rate of grade I-II cardiovascular complications compared to the surgery-alone group (*P*=0.003).

**Table 2:** Postoperative complications assessed according to the Clavien-Dindo classification between the surgery-alone group and the nIComT group.

Postoperative complications	Surgery-alone Group (*n*=116)	The nIComT Group (*n*=116)	*P* value
No. of Patients who experienced any grade of complications	60 (51.7%)	65 (56.0%)	.510
Pulmonary	28 (24.1%)	34 (29.3%)	.373
Cases	36	53	.303
Grade I-II	21	28	.383
≥Grade III	15	25	.224
Cardiac	42 (36.2%)	66 (56.9%)	.002
Cases	53	79	.002
Grade I-II	52	77	.003
≥Grade III	1	2	.562
Gastrointestinal	42 (36.2%)	43 (37.1%)	.892
Cases	46	56	.602
Grade I-II	38	43	.551
≥Grade III	8	13	.342
Urologic	0 (0.0%)	2 (1.7%)	.478
Cases	0	2	.156
Grade I-II	0	2	.156
Thromboembolic	0 (0.0%)	2 (1.7%)	.478
Cases	0	2	.156
Grade I-II	0	2	.156
Neurologic/Psychiatric	5 (4.3%)	3 (2.6%)	1.000
Cases	5	3	.473
Grade I-II	5	2	.409
≥Grade III	0	1	.317
Infection	4 (3.4%)	3 (2.6%)	1.000
Cases	4	3	.702
Grade I-II	0	2	.156
≥Grade III	4	1	.176
Other complications	4 (3.4%)	4 (3.4%)	1.000
Cases	4	4	1.000
Grade I-II	4	3	.702
≥Grade III	0	1	.317

nIComT, neoadjuvant immunotherapy combination treatments.

During the postoperative period, 147 complications occurred in the surgery-alone group versus 202 in the nIComT group (*P*=0.026). Rates of common complications including respiratory, gastrointestinal, urologic, thromboembolic, neuropsychiatric, and infectious were similar (*P*>0.05). Hypotension was significantly more frequent in the nIComT group (52.6% *vs* 42.2%, *P*=0.004), managed via continuous vasopressor including dopamine, ephedrine, norepinephrine, or transfusion. Although not statistically significant, the nIComT group underwent a trend of higher rate of heart failure (10.3% *vs* 4.3%, *P*=0.078) compared with the surgery-alone group. One nIComT patient required ICU readmission for hemorrhagic shock within 48 hours; another developed grade 3 immune-related myocarditis one month postoperatively, presenting with elevated creatine kinase levels not observed during neoadjuvant therapy. **Table 3** presents the incidences of complications of each system.

**Table 3:** Postoperative complications between the surgery-alone group and the nIComT group.

Postoperative complications	Surgery-alone Group (*n*=116)	The nIComT Group (*n*=116)	*P* value
Total number of cases of complications	147	202	.026
Pulmonary			
Pneumonia	21 (18.1%)	26 (22.4%)	.414
Pleural effusion requiring additional drainage procedure	3 (2.6%)	6 (5.2%)	.497
Pneumothorax requiring intervention	2 (1.7%)	4 (3.4%)	.679
Atelectasis mucous plugging requiring bronchoscopy	8 (6.9%)	10 (8.6%)	.624
Respiratory failure requiring reintubation	2 (1.7%)	5 (4.3%)	.443
Acute aspiration	0 (0.0%)	2 (1.7%)	.478
Cardiac			
Arrhythmias requiring intervention	8 (6.9%)	4 (3.4%)	.236
Hypotension requiring intervention	49 (42.2%)	61 (52.6%)	.004
Myocarditis	0 (0.0%)	1 (0.9%) ^a^	1.000
Cardiac arrest requiring CPR	1 (0.9%)	1 (0.9%)	1.000
Heart failure requiring intervention	5 (4.3%)	12 (10.3%)	.078
Gastrointestinal			
Anastomotic leak	12 (10.3%)	13 (11.2%)	1.000
GI bleeding requiring intervention or transfusion	0 (0.0%)	1 (0.9%)	1.000
Small bowel obstruction	1 (0.9%)	0 (0.0%)	1.000
Liver dysfunction	33 (28.4%)	42 (36.2%)	.206
Urologic			
Acute renal insufficiency	0 (0.0%)	2 (1.7%)	.478
Thromboembolic			
PE	0 (0.0%)	1 (0.9%)	1.000
DVT	0 (0.0%)	1 (0.9%)	1.000
Neurologic/Psychiatric			
Recurrent nerve injury	4 (3.4%)	2 (1.7%)	.679
Other neurologic injury	0 (0.0%)	1 (0.9%)	1.000
Infection			
Wound infection requiring opening wound or antibiotics	4 (3.4%)	2 (1.7%)	.679
Central IV-line infection requiring removal or antibiotics	0 (0.0%)	1 (0.9%)	1.000
Other complications			
Chyle leak	4 (3.4%)	3 (2.6%)	1.000
Hemorrhagic shock	0 (0.0%)	1 (0.9%)	1.000

a The patient had immune-mediated myocarditis.

nIComT, neoadjuvant immunotherapy combination treatments; CPR, cardiopulmonary resuscitation; PE, pulmonary embolus; DVT, deep venous thrombosis.

Compared to the nICT subgroup, the nICRT subgroup exhibited significantly higher overall complication rates (47.9% *vs* 95.0%, *P*<0.001), primarily driven by cardiovascular complications (54.2% *vs* 65.0%, *P*=0.068). Specifically, compared with the nICT subgroup, intervention-requiring heart failure was more frequent in the nICRT subgroup (7.3% *vs* 25.0%, *P*=0.050), with a trend toward increased pneumonia (18.8% *vs* 40.0%, *P*=0.075). In addition, the nICRT subgroup had longer operative durations, greater perioperative blood loss, increased intraoperative urine output, prolonged hospital stays, and higher costs than the nICT subgroup (*P*<0.05). Detailed data are provided in **Supplementary Tables S1-S3**.

### Other perioperative outcomes and mortality

**Table 4** demonstrates the TRAEs that occurred in the nIComT group during neoadjuvant treatment. The most common TRAEs in the nIComT group were anemia (*n*=74, 63.8%), lymphocytopenia (*n*=72, 62.1%), and thrombocytopenia (*n*=42, 36.2%). Compared with the nICT subgroup, the nICRT subgroup exhibited significantly higher incidence rates of lymphocytopenia (54.2% *vs* 100.0%), thrombocytopenia (29.2% *vs* 70.0%), leukopenia (19.8% *vs* 75.0%), neutropenia (14.6% *vs* 80.0%), and elevated lactate dehydrogenase (13.5% *vs* 45.0%) (*P*<0.05). Notably, all 20 patients in the nICRT subgroup developed lymphocytopenia (100%). No irAEs were observed in the nIComT group during neoadjuvant treatment.

**Table 4:** TRAEs during neoadjuvant treatment in the nIComT group.

TRAEs	The nIComT group (*n*=116)	The nICT group (*n*=96)	The nICRT group (*n*=20)	*P* value
Anemia	74 (63.8%)	59 (61.5%)	15 (75.0%)	.252
Lymphocytopenia	72 (62.1%)	52 (54.2%)	20 (100.0%)	<0.001
Thrombocytopenia	42 (36.2%)	28 (29.2%)	14 (70.0%)	<0.001
Leukopenia	34 (29.3%)	19 (19.8%)	15 (75.0%)	<0.001
Neutropenia	30 (25.9%)	14 (14.6%)	16 (80.0%)	<.001
Elevated creatine kinase isoenzyme	26 (22.4%)	21 (21.9%)	5 (25.0%)	.992
Elevated creatine kinase	12 (10.3%)	10 (10.4%)	2 (10.0%)	1.000
Elevated BNP-pro	11 (9.5%)	9 (9.4%)	2 (10.0%)	1.000
Elevated CK-MB	4 (3.4%)	3 (3.1%)	1 (5.0%)	1.000
Elevated lactate dehydrogenase	22 (19.0%)	13 (13.5%)	9 (45.0%)	.003
Elevated γ-glutamyltransferase	40 (34.5%)	32 (33.3%)	8 (40.0%)	.568
Elevated aspartate aminotransferase	11 (9.5%)	8 (8.3%)	3 (15.0%)	.613
Elevated alanine aminotransferase	18 (15.5%)	15 (15.6%)	3 (15.0%)	1.000
Elevated direct bilirubin	21 (18.1%)	16 (16.7%)	5 (25.0%)	.575
Elevated total bilirubin	16 (13.8%)	11 (11.5%)	5 (25.0%)	.215
Hypoalbuminemia	15 (12.9%)	11 (11.5%)	4 (20.0%)	.503
Hypothyroidism	10 (8.6%)	6 (6.3%)	4 (20.0%)	.120
Hyperthyroidism	5 (4.3%)	3 (3.1%)	2 (10.0%)	.440
Hyperglycemia	17 (14.7%)	14 (14.6%)	3 (15.0%)	1.000
Hyponatremia	4 (3.4%)	4 (4.2%)	0 (0.0%)	.798
Hypokalemia	15 (12.9%)	10 (10.4%)	5 (25.0%)	.161

TRAEs, Treatment-related adverse event; nIComT, neoadjuvant immunotherapy combination treatments; nICT, neoadjuvant immunotherapy combined with chemotherapy; nICRT, neoadjuvant immunotherapy combined with chemoradiotherapy.

The other perioperative outcomes between the two groups were summarized in **Table 5**. The mean operative duration was significantly longer in the nIComT group, when compared with the surgery-alone group (276 ± 66min *vs* 246 ± 63min, *P*<0.001). A higher proportion of nIComT patients required intraoperative vasopressors including dopamine, ephedrine, norepinephrine (52.6% *vs* 33.6%, *P*=0.004) and perioperative transfusions (34.5% *vs* 14.7%, *P*<0.001). Postoperative thoracic drainage volume (*P*=0.071) and ICU readmission rates (9.5% *vs* 3.4%, *P*=0.062) showed increasing trends in the nIComT group, though non-significant. No other perioperative outcomes differed significantly between groups.

**Table 5:** Perioperative outcomes between the surgery-alone group and the nIComT group.

Perioperative outcomes	Surgery-alone group (*n*=116)	The nIComT group (*n*=116)	*P* value
Operation duration (min, mean ± SD)	246 ± 63	276 ± 66	<.001
Intraoperative use of vasopressor medications (n)	39 (33.6%)	61 (52.6%)	.004
Intraoperative transfusion (n)	3 (2.6%)	7 (6.0%)	.196
Perioperative transfusion (n)	17 (14.7%)	40 (34.5%)	<.001
Intraoperative blood loss (mL, mean ± SD)	173 ± 115	200 ± 226	.338
Intraoperative urine output (mL, mean ± SD)	395 ± 249	426 ± 245	.201
Postoperative thoracic drainage (days, mean ± SD)	8 ± 4	9 ± 4	.071
Postoperative hospital stays (days, mean ± SD)	15 ± 12	15 ± 9	.203
Hospital cost (10000RMB, mean ± SD)	9.62 ± 2.02	10.07 ± 2.36	.183
ICU readmission (n)	4 (3.4%)	11 (9.5%)	.062
30-day readmission (n)	39 (33.6%)	39 (33.6%)	1.000
30-day mortality (n)	1 (0.9%)	2 (1.7%)	1.000

nIComT, neoadjuvant immunotherapy combination treatments; SD, standard deviation.

One patient in the surgery-alone group died within 48 hours after surgery due to respiratory cardiac arrest resulting from the development of atrial fibrillation complicated by respiratory failure. Two patients in the nIComT group died within 30 days of surgery, including one patient received nICT (cardiac arrest due to hypotension with sinus tachycardia within 24 hours) and one patient received nICRT (metabolic encephalopathy with secondary epilepsy). No significant difference in mortality was observed between groups (*P*>0.05).

### Relative factors associated with cardiovascular complications

**Figure 3:**
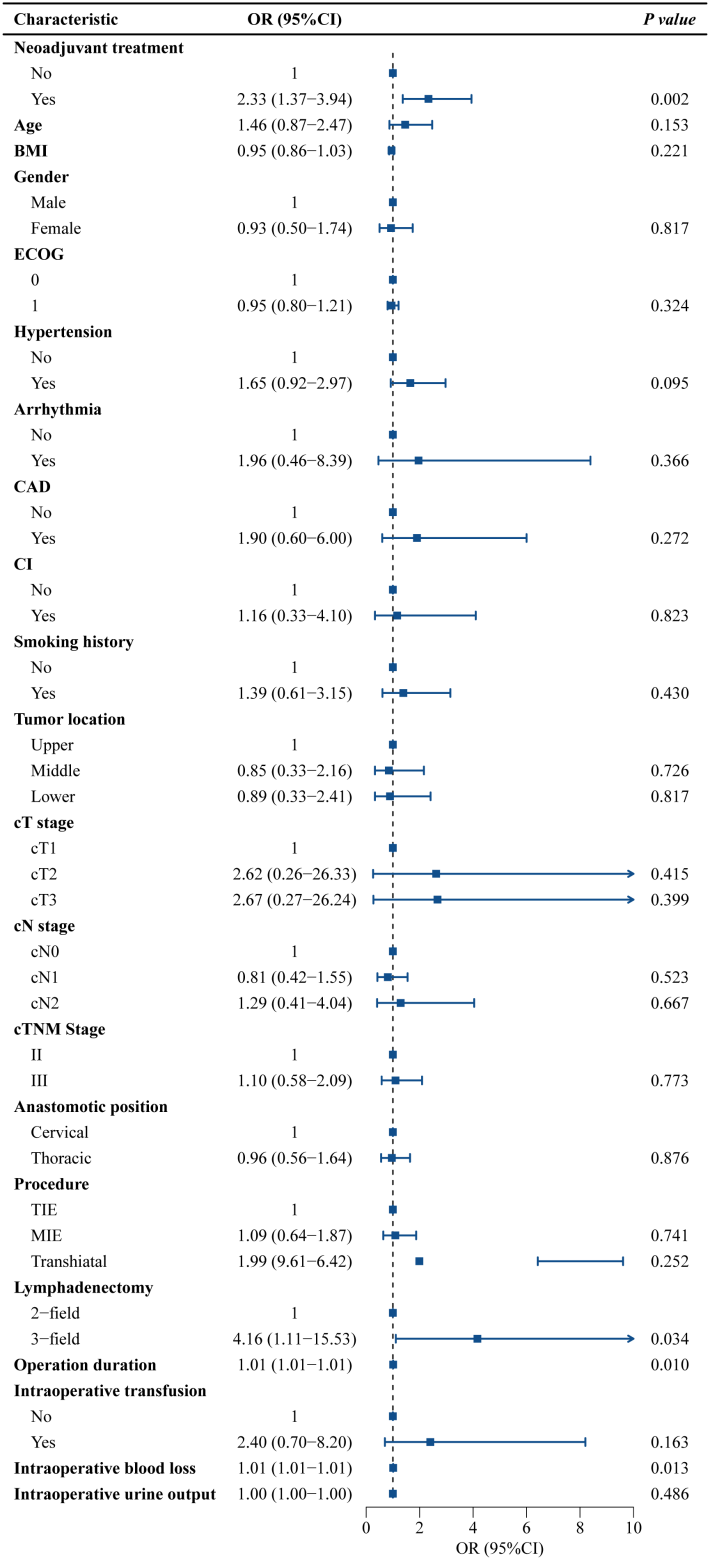
Univariate logistic regression analyses of potential risk factors for cardiovascular complications. OR, odds ratio; 95% CI, 95% confidence interval; BMI, body mass index; ECOG, Eastern Cooperative Oncology Group; CAD, coronary artery disease; CI, cerebral infarction; T, tumor; Tis, carcinoma *in situ*; N, node; TIE, transmediastinal esophagectomy; MIE, minimally invasive esophagectomy.

**Figure 4:**
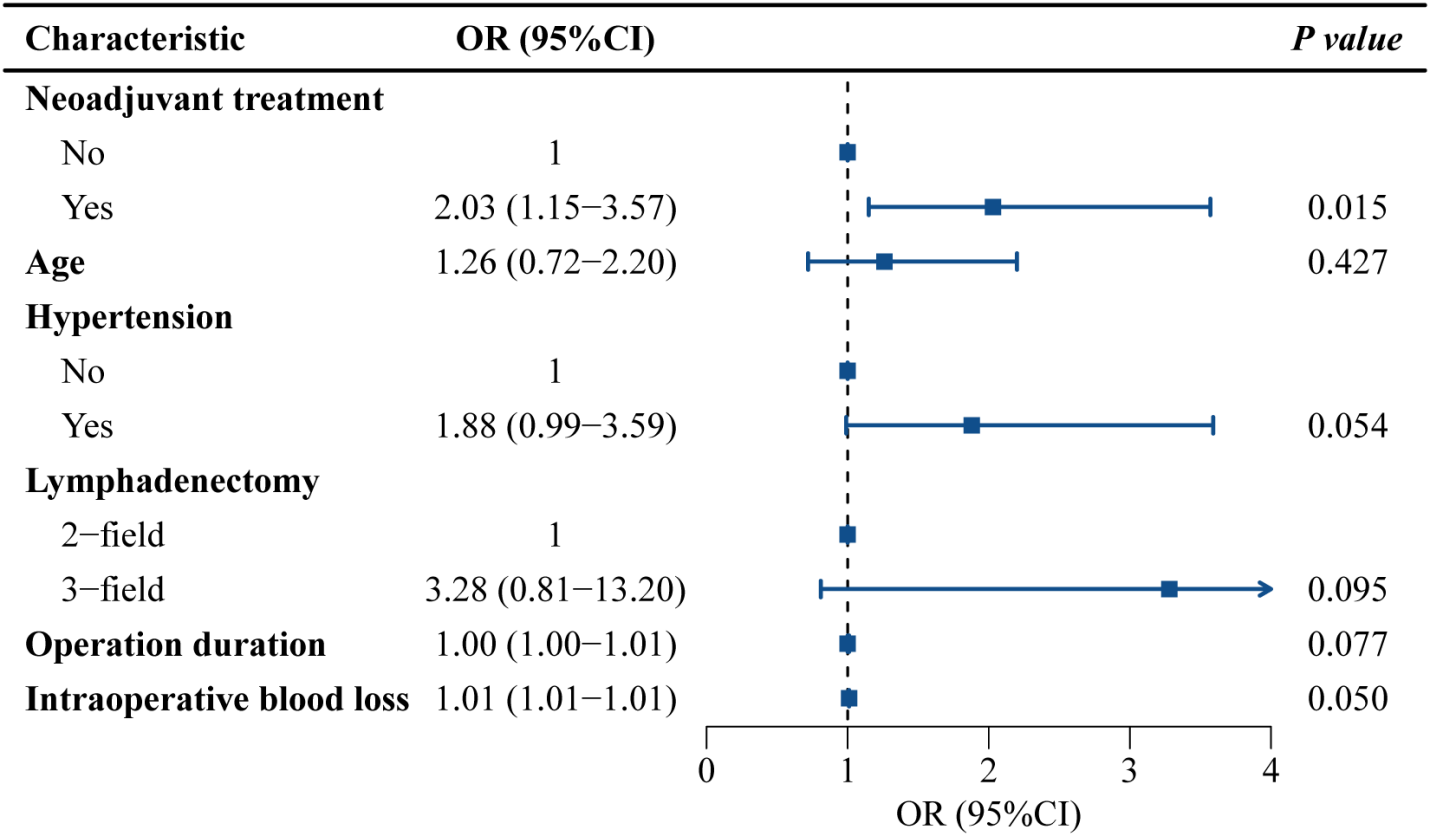
Multivariate logistic regression analyses of potential risk factors for cardiovascular complications. OR, odds ratio; 95% CI, 95% confidence interval.

**Figures 3**, **4** present the univariate and multivariate logistic regression analyses of potential risk factors for cardiovascular complications. Univariate analysis identified significant associations with neoadjuvant therapy (OR=2.33, 95% CI=1.37-3.94, *P*=0.002), three-field lymphadenectomy (OR=4.16, 95% CI=1.11-15.53, *P*=0.034), operative duration (OR=1.01, 95% CI=1.01-1.01, *P*=0.010), and intraoperative blood loss (OR=1.01, 95% CI=1.01-1.01, *P*=0.013). Multivariate analysis demonstrated that neoadjuvant therapy remained independently associated with cardiovascular complications (OR=2.03, 95% CI=1.15-3.57, *P*=0.015).

## Discussion

There is no consistent conclusion in whether nIComT increase postoperative toxicity in LA-ESCC patients. In this study, we retrospectively compared the occurrence of postoperative complications in the surgery-alone group versus nIComT group. Our findings revealed that both groups exhibited similar complication rates across systems, except for the cardiovascular system. Notably, the increasing occurrence of grade I-II cardiovascular complications was reported for the first time in the present study, underscoring the significance of postoperative circulatory interventions for patients who underwent nIComT.

As previously reported, the rates of overall systemic complications were not statistically different from the surgery-alone group as compared with neoadjuvant chemotherapy (nCT) or nCRT group (15, 30). A study comparing esophagectomy after nCT with surgery directly demonstrates that the occurrence of postoperative complications is similarly between the two groups of patients (30). Furthermore, the CROSS study also demonstrates that the incidence of postoperative complications, including those related to the cardiovascular system, is comparable between patients who received esophagectomy following nCRT and those who underwent surgery directly (15). Additionally, several studies with small sample sizes have demonstrated the acceptable safety profile of nICT or nICRT in the treatment of LA-ESCC (31–38). Consistent with previous studies, the nIComT group in our research also indicates that comparable safety profile of systemic complications and severe complications of grade III or above. However, our study is the first to discover that postoperative cardiovascular complications of grade I-II occur significantly more frequently in the nIComT group than in the control group, primarily manifesting as hypotension requiring treatment and heart failure. The NEOCRTEC5010 study also found that the nCRT group had a higher incidence of cardiovascular system complications compared to the surgery-alone group (16), but mainly arrhythmias. In conclusion, despite reports of the safety of neoadjuvant treatment, the occurrence of postoperative hypotension has received limited attention. Our study examined hypotension as part of postoperative cardiovascular complications and identified an increased risk associated with nIComT. Therefore, it is imperative to monitor for hypotension in patients receiving nIComT postoperatively.

The underlying causes of postoperative hypotension in non-cardiac surgery include decreased cardiac output due to hypovolemia, cardiac pump failure or obstruction, and decreased vascular volume resulting from inflammation, pharmacologic interventions, or sympathetic nervous system compromise (39). It was observed that esophagectomy is susceptible to hypotension and arrhythmias due to atrial pressure and cardiac rotation resulting from mediastinal maneuvers, as well as vagus nerve stimulation (40). However, it is noteworthy that there is a significant difference in the incidence of hypotension between the surgery-alone and the nIComT group as reported by the present study. Therefore, the combination of neoadjuvant treatment may be the primary factor contributing to the increased incidence of hypotension in the nIComT group.

Neoadjuvant treatment including chemotherapy, chemoradiotherapy, and immunotherapy, all can cause damage to the heart (41). Through cytotoxicity-induced myocardial damage, chemotherapy can lead to a variety of cardiovascular system complications, such as systolic and diastolic dysfunction of the heart (42). It is also undeniable that microvascular and macrovascular damage may result from ionizing radiation exposure following chest radiation therapy, which can subsequently lead to conditions such as pericarditis, myocardial injury, and myocardial ischemia (43). ICIs can cause systemic multi-system inflammation (44). Studied have demonstrated that ICIs can cause structural changes in the heart, leading to myocardial edema and apical ballooning (45). The clinical manifestations of its cardiotoxicity are highly variable, ranging from asymptomatic elevations of cardiac biomarkers to the rapid onset of cardiogenic shock, including hemodynamic failure due to myocarditis (46). A study specifically exploring the impact of nCT and nCRT on postoperative cardiac complications in LA-ESCC patients found that nCRT posed a greater risk of cardiac system complications than nCT, particularly in terms of N-terminal pro-B-type natriuretic peptide (NT-proBNP) elevation (47). Although the differences in the study did not reach statistical significance, the incidence of arrhythmia reported for nCT and nCRT in the study was 19% and 25.5%, while the incidence rate of heart failure was 6.3% and 3.5%, respectively (47). In the CROSS and NEOCRTE5010 studies, the rates among patients receiving nCRT were 21% and 14.1%, respectively (15, 16). In the nIComT group of our study, the overall incidence of cardiovascular complications reached 15.5%, with a notably higher rate of 12.4% of patients received nICT and 30.0% in patients received nICRT. The incidence of cardiovascular complications in subgroup analysis of our study was comparable or higher than that reported in previous studies. Meanwhile, studies have also reported that the combining ICIs with neoadjuvant chest radiation therapy not only improves the prognosis for patients with non-small cell lung cancer (NSCLC), but also increases the incidence of cardiovascular system complications (48), although the mechanism is not fully understood. The addition of ICIs may be a key factor contributing to the increased risk of postoperative cardiovascular complications in the nIComT group. In conclusion, the integration of neoadjuvant immunotherapy with nCT or nCRT holds the potential to synergistically exacerbate postoperative cardiovascular injury. Consequently, the toxicity associated with nIComT can be mitigated by regulating its intensity, including reducing the dosage of chemotherapy or chemoradiotherapy. Moreover, patients underwent nIComT require heightened vigilance towards the toxicity of the cardiovascular system throughout the postoperative period.

Hypotension was defined as MAP <60 mmHg, consistent with thresholds associated with adverse outcomes in non-cardiac surgery (49). Although intraoperative hemodynamic management remains controversial (50–53), our findings demonstrate significant clinical relevance: 42.2% of surgery-alone patients and 52.6% of nIComT patients required vasopressors for hypotension – a higher incidence than historical esophagectomy cohorts (54). This elevated vasopressor demand in the nIComT group likely reflects cardiovascular stress from synergistic effects of ICIs combined with chemotherapy or radiotherapy, consistent with reported T-cell-mediated myocardial injury mechanisms by PD-1 inhibitors (55). Furthermore, patients with a history of nIComT use, especially those who have received nICRT, should be monitored for vital sign changes during the perioperative period, and the occurrence of serious complications should be recognized and evaluated in a timely manner. Furthermore, postoperative hypotension can lead to oliguria, pulmonary edema, respiratory failure and other adverse consequences. Therefore, in the prospectively study currently conducted in our center, we have adopted intravenous infusion of vasoactive drugs such as norepinephrine for 3–7 days after esophagectomy to maintain blood pressure close to the preoperative normal level, and have initially achieved good results.

This study has several limitations. Firstly, as a retrospective analysis with a small sample size, it carries inherent risks of selection bias and therapeutic heterogeneity. Secondly, the underrepresentation of the nCRT cohort reflects real-world clinical patterns in China, where most LA-ESCC patients with advanced diagnoses and compromised performance status preferentially receive nCT. Lastly, the lack of systematic perioperative cardiac function parameters in some cases hindered comprehensive assessment of treatment-related cardiotoxicity. Future studies should prioritize prospective clinical trials implementing standardized protocols, incorporating comprehensive cardiovascular monitoring and mechanistic investigations to validate these findings. Future prospective studies are needed to validate these findings.

## Data Availability

The original contributions presented in the study are included in the article/**Supplementary Material**. Further inquiries can be directed to the corresponding authors.
