# Supplementary material for: Impact of neoadjuvant immunotherapy combined with chemotherapy or chemoradiotherapy on postoperative safety in locally advanced esophageal squamous cell carcinoma: a propensity score-matched retrospective cohort study
Source: Front Oncol. 2025 May 21;15:1573597. doi: 10.3389/fonc.2025.1573597 (PMC12133542; doi:10.3389/fonc.2025.1573597)
Supplement: Supplementary file 1 [file Table1.docx]

**Table S1. Comparison of perioperative complications according to the Clavien-Dindo classification between nICT and nICRT group**

|  | **The nICT Group (*n*=96)** | **The nICRT Group (*n*=20)** | ***P* value** |
| --- | --- | --- | --- |
| **No. of Patients who experienced any grade of complications** | 46 (47.9%) | 19 (95.0%) | <.001 |
| **Pulmonary** | 26 (27.1%) | 8 (40.0%) | .248 |
| Cases | 45 | 8 | .576 |
| Grade Ⅰ-Ⅱ | 24 | 4 | .636 |
| ≥Grade Ⅲ | 21 | 4 | .853 |
| **Cardiac** | 52 (54.2%) | 13 (65.0%) | .068 |
| Cases | 59 | 20 | .069 |
| Grade Ⅰ-Ⅱ | 57 | 20 | .056 |
| ≥Grade Ⅲ | 2 | 0 | .497 |
| **Gastrointestinal** | 34 (35.4%) | 9 (45.0%) | .420 |
| Cases | 43 | 13 | .165 |
| Grade Ⅰ-Ⅱ | 34 | 11 | .173 |
| ≥Grade Ⅲ | 9 | 2 | .267 |
| **Urologic** | 2 (2.1%) | 0 (0.0%) | 1.000 |
| Cases | 2 | 0 | .497 |
| Grade Ⅰ-Ⅱ | 2 | 0 | .497 |
| **Thromboembolic** | 2 (2.1%) | 0 (0.0%) | 1.000 |
| Cases | 2 | 0 | .497 |
| Grade Ⅰ-Ⅱ | 2 | 0 | .497 |
| **Neurologic/Psychiatric** | 3 (3.1%) | 0 (0.0%) | .929 |
| Cases | 3 | 0 | .403 |
| Grade Ⅰ-Ⅱ | 2 | 0 | .497 |
| ≥Grade Ⅲ | 1 | 0 | .632 |
| **Infection** | 3 (3.1%) | 0 (0.0%) | .929 |
| Cases | 3 | 0 | .403 |
| Grade Ⅰ-Ⅱ | 2 | 0 | .497 |
| ≥Grade Ⅲ | 1 | 0 | .632 |
| **Other complications** | 4 (4.2%) | 0 (0.0%) | .748 |
| Cases | 4 | 0 | .332 |
| Grade Ⅰ-Ⅱ | 3 | 0 | .403 |
| ≥Grade Ⅲ | 1 | 0 | .632 |

nICT, neoadjuvant immunotherapy combined with chemotherapy; nICRT, neoadjuvant immunotherapy combined with chemoradiotherapy

**Table S2. Post-operative complications between nICT and nICRT group**

|  | **The nICT Group (*n*=96)** | **The nICRT Group (*n*=22)** | ***P* value** |
| --- | --- | --- | --- |
| **Total number of cases of complications** | 161 | 41 | .037 |
| **Pulmonary** |  |  |  |
| Pneumonia | 18 (18.8%) | 8 (40.0%) | .075 |
| Pleural effusion requiring additional drainage procedure | 5 (5.2%) | 1 (5.0%) | 1.000 |
| Pneumothorax requiring intervention | 4 (4.2%) | 0 (0.0%) | .798 |
| Atelectasis mucous plugging requiring bronchoscopy | 9 (9.4%) | 1 (5.0%) | .844 |
| Respiratory failure requiring reintubation | 4 (4.2%) | 1 (5.0%) | 1.000 |
| Acute aspiration | 2 (2.1%) | 0 (0.0%) | 1.000 |
| **Cardiac** |  |  |  |
| Ventricular dysrhythmia requiring intervention | 3 (3.1%) | 1 (5.0%) | 1.000 |
| Hypotension with sinus tachycardia requiring intervention | 47 (49.0%) | 14 (70.0%) | .086 |
| Myocarditis | 1 (1.0%) | 0 (0.0%) | 1.000 |
| Cardiac arrest requiring CPR | 1 (1.0%) | 0 (0.0%) | 1.000 |
| Congestive heart failure requiring intervention | 7 (7.3%) | 5 (25.0%) | .050 |
| **Gastrointestinal** |  |  |  |
| Anastomotic leak | 10 (10.4%) | 3 (15.0%) | .840 |
| GI bleeding requiring intervention or transfusion | 1 (1.0%) | 0 (4.5%) | 1.000 |
| Liver dysfunction | 32 (33.3%) | 10 (50.0%) | .158 |
| **Urologic** |  |  |  |
| Acute renal insufficiency | 2 (2.1%) | 0 (0.0%) | 1.000 |
| **Thromboembolic** |  |  |  |
| PE | 1 (1.0%) | 0 (0.0%) | 1.000 |
| DVT | 1 (1.0%) | 0 (0.0%) | 1.000 |
| **Neurologic/Psychiatric** |  |  |  |
| Recurrent nerve injury | 2 (2.1%) | 0 (0.0%) | 1.000 |
| Other neurologic injury | 1 (1.0%) | 0 (0.0%) | 1.000 |
| **Infection** |  |  |  |
| Wound infection requiring opening wound or antibiotics | 2 (2.1%) | 0 (0.0%) | 1.000 |
| Central IV-line infection requiring removal or antibiotics | 1 (1.0%) | 0 (0.0%) | 1.000 |
| **Other complications** |  |  |  |
| Chyle leak | 3 (3.1%) | 0 (0.0%) | .979 |
| Hemorrhagic shock | 1 (1.0%) | 0 (0.0%) | 1.000 |

nICT, neoadjuvant immunotherapy combined with chemotherapy; nICRT, neoadjuvant immunotherapy combined with chemoradiotherapy; CPR, cardiopulmonary resuscitation; PE, pulmonary embolus; DVT, deep venous thrombosis.

**Table S3. Perioperative outcomes between nICT and nICRT group**

|  | **The nICT Group (*n*=96)** | **The nICRT Group (*n*=22)** | ***P* value** |
| --- | --- | --- | --- |
| Operation duration (min, mean ± SD) | 261±59 | 350±44 | <.001 |
| Intraoperative use of vasopressor medications (n) | 45 (46.9%) | 16 (80.0%) | .007 |
| Intraoperative transfusion (n) | 7 (7.3%) | 0 (0.0%) | .466 |
| Perioperative transfusion (n) | 26 (27.1%) | 14 (70.0%) | <.001 |
| Intraoperative blood loss (mL, mean ± SD) | 203±245 | 174±101 | .884 |
| Intraoperative urine output (mL, mean ± SD) | 392±216 | 564±311 | .014 |
| Postoperative thoracic drainage (days, mean ± SD) | 9±4 | 9±4 | .293 |
| Postoperative hospital stay (days, mean ± SD) | 14±9 | 19±10 | <.001 |
| Hospital cost (10000RMB, mean ± SD) | 9.76±2.39 | 11.87±2.37 | <.001 |
| ICU readmission (n) | 9 (9.4%) | 2 (10.0%) | 1.000 |
| 30-day readmission (n) | 34 (35.4%) | 5 (25.0%) | .370 |
| 30-day mortality (n) | 1 (1.0%) | 1 (5.0%) | .770 |
|  |  |  |  |

nICT, neoadjuvant immunotherapy combined with chemotherapy; nICRT, neoadjuvant immunotherapy combined with chemoradiotherapy; SD, standard deviation
